# The Role of Conization before Radical Hysterectomy in Cervical Cancer including High Risk Factors of Recurrence: Propensity Score Matching

**DOI:** 10.3390/cancers14163863

**Published:** 2022-08-10

**Authors:** Chi-Son Chang, Ji Song Min, Ki Hyeon Song, Chel Hun Choi, Tae-Joong Kim, Jeong-Won Lee, Byoung-Gie Kim, Yoo-Young Lee

**Affiliations:** Department of Obstetrics and Gynecology, Samsung Medical Center, Sungkyunkwan University School of Medicine, Seoul 06351, Korea

**Keywords:** conization, cervical cancer, radical hysterectomy, prognosis, propensity score matching

## Abstract

**Simple Summary:**

We investigated the therapeutic role of conization prior to radical hysterectomy for stage IB1 to IIB cervical cancer regardless of pathologic high risk factors, using the propensity score matching method. Additional subgroup analysis was carried out to identify the effect of conization based on clinical and pathologic factors. Conization before radical hysterectomy was associated with reduced recurrence and mortality. Conization had benefit in patients with MIS, negative pelvic lymph nodes, tumor sized less than 4 cm. Delays of definite treatment due to conization did not have an effect on the prognosis of patients with high risk factors. This study reveals the protective role of preoperative conization in early-stage cervical cancer, along with identifying subgroups that may best benefit from the procedure. This information can be used for more tailored patient selection for minimally invasive surgery for cervical cancer.

**Abstract:**

We primarily aimed to investigate the therapeutic role of conization prior to radical hysterectomy for cervical cancer. Secondarily, we aimed to characterize a subgroup of patients who could potentially benefit from preoperative conization. Patients who underwent radical hysterectomy for FIGO 2009 stage IB1 to IIB cervical cancer from 1995 to 2020 were eligible. The patients were divided into two groups: those with and without preoperative conization. To adjust for the baseline characteristics of the two groups, 1:2 case–control propensity score matching was conducted. Survival analysis was performed between the two groups. Subgroup analysis was performed to identify the effect of conization based on clinical and pathological factors. Patients who underwent preoperative conization showed better 5-year overall survival than those who did not (95.9% vs. 93.0%, *p* = 0.029); however, no difference was observed in progression-free survival (88.9% vs. 85.9%, *p* = 0.155). In multivariate Cox analysis, conization showed a 55% reduction in recurrence (hazard ratio (HR) 0.65, 95% confidence interval (CI) 0.41–1.01, *p* = 0.056) and 41% reduction in death (HR 0.59, 95% CI 0.34–1.02, *p* = 0.059), but with marginal statistical significance. In subgroup analysis, minimally invasive surgery (MIS), negative pelvic lymph node, and tumor size < 4 cm showed improved survival from conization. Conization before radical hysterectomy may be associated with improved survival in patients with early-stage cervical cancer. This information could serve as a basis for a more tailored patient selection for MIS for cervical cancer.

## 1. Introduction

Even after the introduction of cervical screening tests and human papillomavirus vaccination, cervical cancer still poses a great burden worldwide, especially in developing countries. It is the fourth most common cancer among women, with 604,127 new cases and 341,831 deaths reported worldwide in 2020 [1]. At the time of diagnosis, more than half of the patients have early-stage cancer [2]. Surgical treatment of cervical cancer is only decided after histological confirmation of cancer via conization or cervical punch biopsy [3]. In such cases, radical hysterectomy with pelvic lymphadenectomy is currently the treatment of choice for stage IA2 to IIA1 disease. Stage IIA2 or IIB cervical cancer may also be treated with upfront radical hysterectomy at the surgeon’s discretion [4].

The treatment role of conization for stages IA1 or IA2 is well established. However, the therapeutic role of conization in early cervical cancer above stage IB1, which is a candidate for radical hysterectomy, has not been investigated. After the publication of LACC (Laparoscopic approach to carcinoma of the cervix) trial in 2018 [5], gynecologic surgeons have been searching for an effective method to reduce tumor spillage, which was suggested to be one of the possible causes for worse prognosis in minimally invasive surgery (MIS) [6,7,8,9,10,11]. Recently, the protective role of preoperative conization has been highlighted in several studies. These studies collectively agreed upon the improved prognosis in patients with preoperative conization [12,13,14,15,16].

However, existing studies that investigated the protective role of conization have been conducted on highly selected patients. In previous reports, most of the patients did not have high- or intermediate-risk factors of Peters and Sedlis criteria [17,18]. However, in real-world clinical practice, patients are upstaged after surgery and required adjuvant radiation therapy (RT) or concurrent chemoradiotherapy (CCRT). It remains unknown whether conization plays a role in these cases. Therefore, we expanded the subject of this study to those who could be considered for radical hysterectomy with or without pathological risk factors.

We primarily aimed to investigate the role of conization before radical hysterectomy in the International Federation of Gynecology and Obstetrics (FIGO) 2009 stage IB1 to IIB cervical cancer. Secondarily, we aimed to characterize a subgroup of patients who could potentially benefit from preoperative conization.

## 2. Materials and Methods

We retrieved medical records of patients with cervical cancer who were treated at a single tertiary academic cancer center between 1995 and 2020 for this retrospective cohort study. Patients who underwent radical hysterectomy for FIGO 2009 stage IB1 to IIB cervical cancer were eligible. Patients with a rare histology or those treated primarily with radiation or chemotherapy were excluded. Approval from the Institutional Review Board (IRB) was obtained prior to data collection (No. 2021-08-058), and informed consent was omitted by IRB owing to the retrospective nature of the study.

Pathological diagnosis of cervical cancer was made either by colposcopy-directed biopsy or conization of the uterine cervix. Conization was performed using the loop electrosurgical excision procedure. Diagnosed patients were treated primarily with radical hysterectomy with or without pelvic/para-aortic lymph node dissection. The surgical approach was determined by the attending physician, open surgery or MIS (i.e., laparoscopy and robotic approach). The total tumor size was determined as the sum of the pathologic diameter of the tumor in the conization and hysterectomy specimens.

Patients with and without high pathological risk factors, such as lymph node metastasis, parametrial invasion, involvement of resection margins, or bulky tumors, were all included in this study. Patients received adjuvant radiotherapy with or without concurrent chemotherapy if they had risk factors. Following treatment, patients underwent follow-up examinations every 3 months for the first 2 years, every 6 months for the next 3 years, and annually thereafter. Progression-free survival (PFS) was assessed from the date of surgery to the date of recurrence or the date of the last follow-up visit. Overall survival (OS) was measured from the date of surgery to the time of death, or for living patients, to the date of the last contact.

Patients were divided into two groups: patients who underwent preoperative conization and those who did not. To adjust for the baseline characteristics in the two groups, 1:2 case–control matching was conducted based on the absolute difference of propensity scores, using greedy nearest matching with caliper of 0.25. Matching was performed on the following variables: age, year of diagnosis, stage, histological type, type of hysterectomy, surgical approach, total tumor size, invasion depth, parametrial invasion, resection margin, lymphovascular space invasion (LVSI), and paraaortic lymph node metastasis.

Statistical analysis was performed using SAS version 9.4 (SAS Institute, Cary, NC, USA) and R 4.1.0 (Vienna, Austria; http://www.R-project.org/ (accessed on 7 October 2021)). For clinical variables, statistical analyses were performed using Student’s t-test or Mann–Whitney U test for continuous data and the Pearson χ^2^ test or Fisher’s exact test for categorical data. Survival distributions were estimated using the Kaplan–Meier method, and the relationship between survival and each parameter was analyzed by the log-rank test. A Cox proportional hazard model based on the robust sandwich covariance matrix was used for both univariable and multivariable analyses. To investigate the role of conization in various subgroups, we estimated the hazard ratios (HR) of recurrence and death that were associated with conization using multivariable models for each subgroup. Subgroups with fewer than 10 recurrences or death events were excluded from the analysis. Statistical significance was considered to be present at values of *p* < 0.05.

## 3. Results

### 3.1. Clinical Characteristics of the Entire Cohort

A total of 1799 patients were eligible in this study. There were 291 patients who underwent preoperative conization (16.2%), and 1508 patients did not undergo conization (83.8%). In the entire cohort, patients who underwent preoperative conization were younger (*p* < 0.001), had a less advanced stage (*p* < 0.001), underwent MIS more frequently (*p* < 0.001), and were less likely to receive adjuvant treatment after surgery (*p* < 0.001). In terms of pathological findings after radical hysterectomy, patients with conization had a smaller tumor size (*p* < 0.001), a larger percentage of squamous cell type (*p* = 0.011), a shallower depth of invasion (*p* < 0.001), and were more likely to have negative LVSI (*p* < 0.001). This group also had less microscopic parametrial invasion (*p* < 0.001) and was less likely to have positive cancer findings in their pelvic or para-aortic lymph nodes (*p* < 0.001 and *p* = 0.042, respectively).

### 3.2. Analysis after Propensity Score Matching

To adjust for the differences in clinical characteristics between the two groups, we performed 1:2 propensity score matching on variables that showed statistical significance, as described above. After matching, 841 patients were included in the analysis comparing 291 patients who had preoperative conization and 550 patients who did not. Clinical and histopathological characteristics were similar between the groups as a result of propensity matched comparison. Table 1 presents the details of patients’ characteristics.

**Figure 1:**
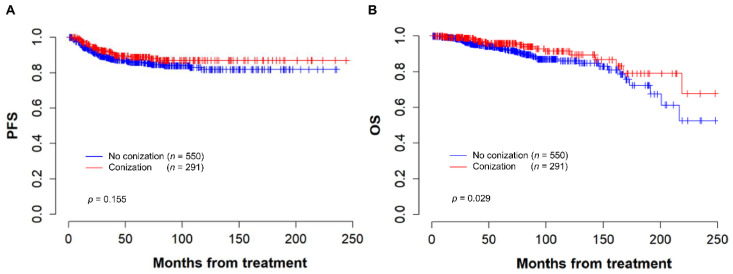
Kaplan–Meier curves for (**A**) progression free survival and (**B**) overall survival based on conization performed before radical hysterectomy.

The median interval between conization and radical surgery was 34 days (range: 27–42.5 days) in the conization group. The median time interval between the first visit to our institution and radical surgery was significantly longer in the conization group than in the control group (37 days, range: 22.0–61.5 days vs. 19 days, range: 12.0–27.0 days, *p* < 0.001). Within a mean follow-up time of 70.4 months, there were 29 (10.0%) cases of recurrence in the conization group, whereas 72 (13.1%) patients developed recurrence in the group without conization (*p* = 0.224). Eighteen (6.2%) patients died in the conization group, whereas 54 (9.8%) patients died in the group without conization (*p* = 0.097). There was no significant difference in the 5-year PFS between patients with and without conization (88.9% vs. 85.9%, *p* = 0.155) (Figure 1A). However, the conization group showed a better 5-year OS than the control group (95.9% vs. 93.0%, *p* = 0.029) (Figure 1B).

The multivariate Cox proportional hazards model showed that the independent prognostic factors for PFS were non-squamous histological type, LVSI, para-aortic lymph node metastasis, and adjuvant treatment after radical surgery. For OS, age, non-squamous histological type, parametrial invasion, and pelvic and para-aortic lymph node metastasis were significant prognostic factors (Table 2). Whether or not conization was performed before radical surgery had marginal significance for both PFS (HR 0.65, 95% confidence interval [CI] 0.41–1.01, *p* = 0.056) and OS (HR 0.59, 95% CI 0.34–1.02, *p* = 0.059). Univariate and multivariate analyses for PFS and OS including all variables showed similar results (Supplementary Tables S1 and S2).

### 3.3. Subgroup Analysis

**Figure 2:**
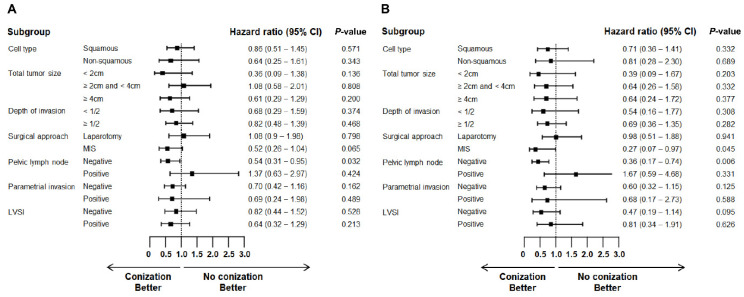
Forest plot of hazard ratio for (**A**) recurrence and (**B**) death in subgroup analysis.

The effect of conization was examined in subgroups stratified by cell type, tumor size, depth of invasion, pelvic lymph node metastasis, parametrial invasion, LVSI, and surgical approach (Figure 2). Overall, preoperative conization was associated with a lower risk of recurrence and death, but not with statistical significance in most subgroups. However, patients who underwent MIS had a reduction in the risk of recurrence (HR 0.52, 95% CI 0.26–1.04, *p* = 0.065) and death (HR 0.27, 95% CI 0.07–0.97, *p* = 0.045) in the conization group compared to the control group. Patients with negative pelvic lymph node metastasis also had significant reduction in the risk of recurrence (HR 0.54, 95% CI 0.31–0.95, *p* = 0.032) and death (HR 0.36, 95% CI 0.17–0.74, *p* = 0.006) in the conization group compared to the control group.

**Figure 3:**
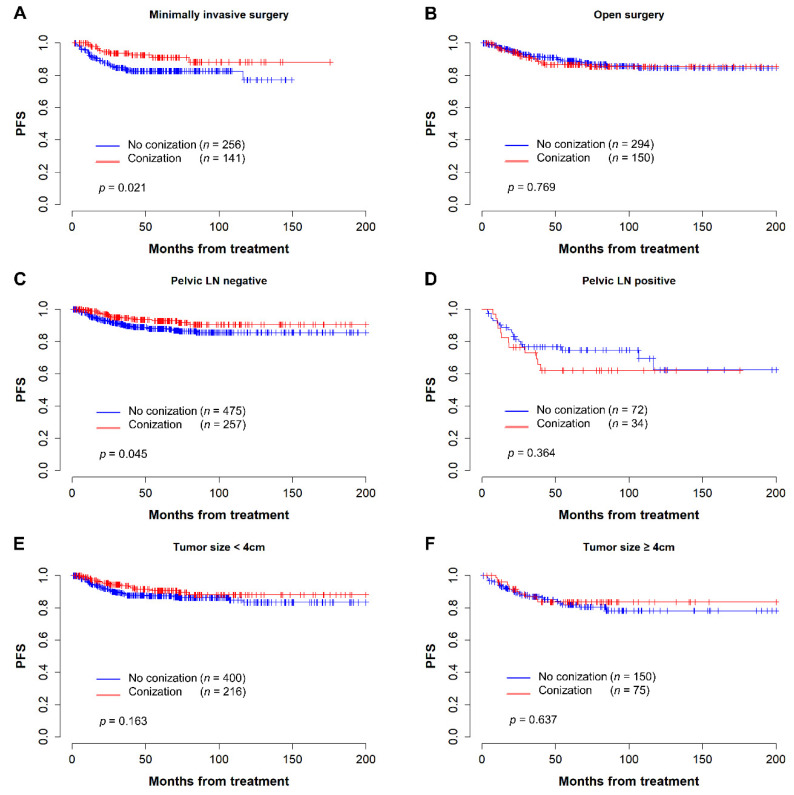
Kaplan–Meier curves for progression-free survival in subgroup analysis. (**A**) Patients who underwent minimally invasive surgery; (**B**) Patients who underwent open surgery; (**C**) Patient without pelvic lymph node metastasis; (**D**) Patients with pelvic lymph node metastasis; (**E**) Patients with tumor size less than 4 cm; (**F**) Patients with tumor size over 4 cm.

**Figure 4:**
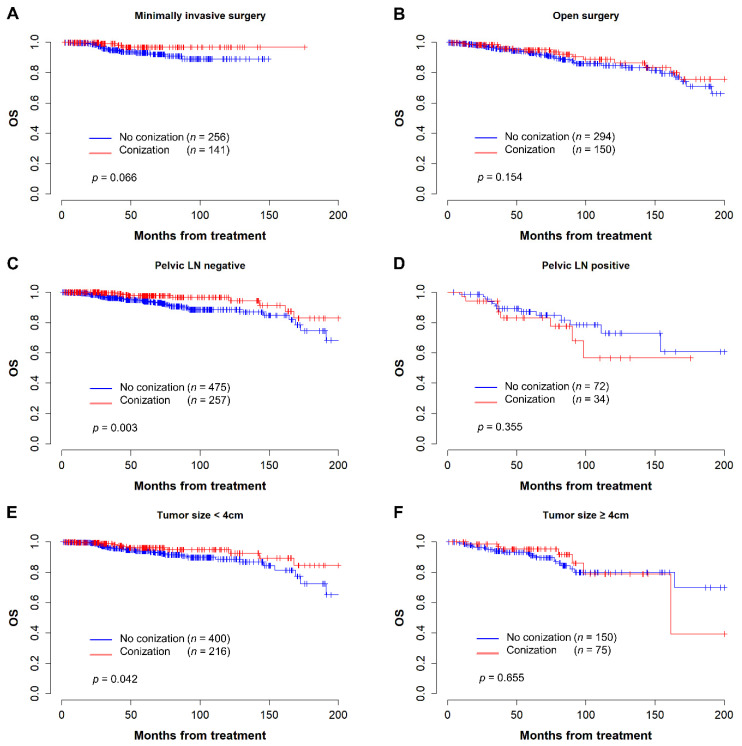
Kaplan–Meier curves for overall survival in subgroup analysis. (**A**) Patients who underwent minimally invasive surgery; (**B**) Patients who underwent open surgery; (**C**) Patient without pelvic lymph node metastasis; (**D**) Patients with pelvic lymph node metastasis; (**E**) Patients with tumor size less than 4 cm; (**F**) Patients with tumor size over 4 cm.

Additional survival analysis was performed by selecting subgroups of three variables: surgical approach, lymph node metastasis, and tumor size. Kaplan–Meier curves demonstrating PFS and OS of conization in each subgroup are shown in Figure 3 and Figure 4, respectively. Among patients who underwent MIS radical hysterectomy, the conization group showed better PFS (5-year PFS, 91.2% vs. 82.7%, *p* = 0.021) and OS (5-year OS 96.9% vs. 93.2%, *p* = 0.066) than the control group (Figure 3A and Figure 4A). In contrast, there was no difference in PFS (5-year PFS, 86.7% vs. 88.9%, *p* = 0.769) or OS (5-year OS, 95.0% vs. 92.9%, *p* = 0.154) for conization in patients who underwent open radical hysterectomy (Figure 3B and Figure 4B). Among patients with negative pelvic lymph nodes, the preoperative conization group had statistically superior PFS (*p* = 0.045) and OS (*p* = 0.003) (Figure 3C and Figure 4C). However, there was no difference in survival for preoperative conization in patients with positive pelvic lymph nodes (Figure 3D and Figure 4D).

Furthermore, we grouped the patients according to the total tumor size. In patients with tumor size less than 4 cm, the conization group showed similar PFS (*p* = 0.163) but superior OS (*p* = 0.042) than the control group (Figure 3E and Figure 4E). However, there was no difference in survival for preoperative conization in patients with tumors larger than 4 cm (Figure 3F and Figure 4F). The trend of a superior OS with conization persisted even when we further split the patients with tumor size less than 4 cm into groups with tumor size < 2 cm and those with tumor size ≥ 2 cm and < 4 cm, but statistical significance was lost (Supplementary Figure S1).

In our matched cohort, 86.3% (251/291) of the conization group had a positive resection margin of conization. Among the three subgroups according to tumor size (<2 cm, ≥2 cm and <4 cm, and ≥4 cm), patients with larger tumor size were more likely to show positive resection margin of conization (81.6% (80/98) vs. 87.3% (103/118) vs. 91.9% (68/75), *p* = 0.049). However, no significant difference in survival was observed between patients with negative or positive conization resection margins (Supplementary Figure S2).

## 4. Discussion

In this study, we investigated the role of conization prior to radical hysterectomy in FIGO 2009 stage IB1 to IIB cervical cancer regardless of pathologic high risk factors, using the propensity score matching method. Conization before radical hysterectomy was associated with improved overall survival. Preoperative conization showed a reduction in recurrence and mortality in multivariate analysis but with marginal statistical significance. In subgroup analysis, better prognosis was observed for preoperative conization in patients who underwent MIS radical hysterectomy, patients with negative pelvic lymph nodes, and patients with tumor size < 4 cm.

The 2018 published LACC trial result showed that MIS had an increased risk of local recurrence and death compared to open surgery [5]. Furthermore, secondary endpoint analyses demonstrated that MIS showed similar perioperative outcomes with open approach, and did not improve postoperative quality of life [19,20]. Thus, updated guidelines including the NCCN and ESGO support that laparotomy should be preferred procedure for radical hysterectomy [4,21].

Researchers have proposed cancer cell contamination as a hypothesis for worse oncologic outcomes in patients with MIS radical hysterectomy [22]. Tumor spillage at the time of intracorporeal colpotomy might contaminate the pelvis, with increased risk of peritoneal carcinomatosis due to CO2 flow [23]. Use of uterine manipulator in MIS was reported to be associated with increased risk of relapse (HR 2.76, 95% CI 1.75–4.33, *p* < 0.001) [8]. Therefore, various methods have been explored to prevent tumor spillage. The SUCCOR (Surgery in Cervical Cancer, Observational, Retrospective) study group reported that MIS without uterine manipulator had similar disease-free survival (DFS) in FIGO 2009 stage IB1 cervical cancer to the open surgery (HR 1.58, 95% CI 0.79–3.15, *p* = 0.20). Additionally, the recurrence rates for patients who underwent MIS with protective vaginal closure and those who underwent open surgery were comparable (HR 0.63, 95% CI 0.15–2.59, *p* = 0.52) [8]. They subsequently reported about significantly lower risk of relapse and death with conization before radical hysterectomy [16,24].

To date, few reports on the role of preoperative conization have been published. Casarin et al. [12] reported that conization before minimally invasive radical hysterectomy in FIGO 2009 stage IA1–IB1 cervical cancer was associated with a lower recurrence rate (1.1% vs. 16.1%, *p* < 0.001). Benoit et al. [13] also drew a similar conclusion showing superior DFS with preoperative conization in patients with FIGO 2018 stage IA1–IB2 cervical cancer, although the result may not be statistically significant (HR 0.19, 95% CI 0.02–1.63, *p* = 0.070). Shortly after, three recent studies investigated the role of conization in early cervical cancer using the propensity score matching method to equalize the characteristics of patients between groups [14,15,16]. They also concluded that preoperative conization improved DFS. In particular, Bizzarri et al. [14] and Kim et al. [15] compared MIS and open surgery separately. They reported no effect of conization in the open surgery group, whereas patients with conization showed improved survival in the MIS group. However, previous studies have been conducted with subjects limited to stage IB1. In our study, we expanded the cohort to include patients with stage IB1 to IIB, and we included advanced stage patients who needed adjuvant treatment after surgery because their final pathology showed high risk factors for recurrence such as positive lymph node metastasis, parametrial invasion, positive resection margins, or bulky tumors.

The major strength of our study is that we stratified the patients into various subgroups to optimize the possible candidates for preoperative conization. Consistent with the results of previous studies [14,15], patients who underwent conization showed better PFS in the MIS group in our study. This result could imply that patients with preoperative conization could be treated safely by MIS without adverse survival outcome. Bogani et al. [25] reported similar DFS (*p* = 0.549) and OS (*p* = 0.615) between open and laparoscopic radical hysterectomy with primary conization in early cervical cancer. They suggested that preoperative conization might overcome the risk of local recurrence after MIS. The probability of tumor leakage might be reduced by tumor excision and size reduction with conization before radical hysterectomy [13,15]. Moreover, improvement in survival with conization was only seen in patients with negative pelvic lymph nodes. This result might be helpful in making decisions regarding the surgical technique that can be performed in these individuals, considering MIS as a reasonable surgical approach with preoperative conization in specific patients.

On the other hand, it was found that preoperative conization has neither a therapeutic nor detrimental role in patients with advanced stage with pelvic lymph node metastasis and bulky tumors over 4 cm in size. Since preoperative conization can delay radical hysterectomy, it could be argued that preoperative conization may be harmful, especially in high tumor volume disease. In our cohort, the median time interval between the first visit to our institution and radical surgery was significantly longer in the conization group than in the control group (37 days vs. 19 days, *p* < 0.001). However, conization did not lead to a poor prognosis in patients with high risk factors. In particular, advanced cervical cancer with lymph node metastasis did not show adverse outcome even when definitive treatment was delayed with conization.

One of the concerns regarding preoperative conization is the lag time between conization and hysterectomy. Due to post-operative complications, such as pelvic infection, some recommend waiting for at least 4 to 6 weeks to perform hysterectomy after conization [26,27]. In contrast, there are reports that showed no change in post-operative morbidity regardless of the time interval between conization and hysterectomy [28,29]. When it comes to the prognosis of cervical cancer, there might be concerns that the disease will progress if the operation is carried out too late. A previous report on the time interval from disease diagnosis to surgery in early cervical cancer showed that a waiting time longer than 8 weeks was associated with worse OS [30]. In contrast, Umezu et al. [31] concluded that a median interval of 48 days before surgery did not affect survival outcomes. Samlal et al. [28] reported no influence on the prognosis of conization prior to surgery in patients with stage IB to IIA cervical cancer when the conization-radical hysterectomy interval was approximately 6 weeks. Furthermore, Benoit et al. [13] reported better DFS with conization as mentioned earlier, with a mean delay between conization and surgical treatment of 43.2 days. In our study, the median interval between conization and radical surgery was 34 days (approximately 5 weeks). Delays of this magnitude did not appear to have an effect on the prognosis of early-stage cervical cancer.

Patients with positive lymph node metastasis eventually require adjuvant treatment, such as radiation with concurrent chemotherapy after radical hysterectomy. In this population, the waiting time until the start of adjuvant treatment after surgery and the duration of chemoradiation are added to complete the initial treatment. There is a consensus that the definition of late initiation of treatment is more than 60 days after diagnosis [32]. This delay is one of the factors that may affect the prognosis of patients with cervical cancer [33,34]. Furthermore, prolonged treatment times from the start to the end of definitive CCRT are associated with worse outcomes for cervical cancer [35,36]. However, we did not investigate the time frame of adjuvant treatment after surgery in this study, which is one of the limitations of our results.

This study has some other limitations. First, this study was mainly based on medical charts, so its retrospective nature might have caused information bias. Second, there were changes in practice (e.g., surgical technique, radiotherapy) over the long-term study period. Third, there is a paucity of detailed information regarding adjuvant treatment, such as the extent of the radiation field or chemotherapy regimens, which can affect prognosis. There is also a need for more research on the impact of conization on recurrence patterns.

## 5. Conclusions

In conclusion, conization before radical hysterectomy may be associated with a reduction in recurrence and mortality in patients with low-risk, early-stage cervical cancer. In particular, patients with a small tumor, < 4 cm, and those with negative pelvic lymph node metastasis benefited from preoperative conization. Conization also showed a protective effect in patients who underwent MIS radical hysterectomy. In addition, no detrimental effect of preoperative conization was observed in high-risk patients. This information could serve as a basis for a more tailored patient selection for MIS for cervical cancer. In addition, it might be helpful to set standards for future prospective studies investigating the role of conization in cervical cancer treatment.

## Figures and Tables

**Table 1:** Patients’ baseline characteristics before and after propensity score matching.

	Unmatched			Matched		
	without Conization (*n* = 1508)	with Conization (*n* = 291)	*p*-Value	without Conization (*n* = 550)	with Conization (*n* = 291)	*p*-Value
Age	48 [16–83]	46 [23–87]	0.001	46 [16–83]	46 [23–87]	0.282
Year of diagnosis			0.009			0.736
~2000	219 (14.52%)	27 (9.28%)		49 (8.91%)	27 (9.28%)	
2000~2010	614 (40.72%)	109 (37.46%)		221 (40.18%)	109 (37.46%)	
2010~	675 (44.76%)	155 (53.26%)		280 (50.91%)	155 (53.26%)	
Stage			<0.001			0.063
IB1 + IB2	1197 (79.38%)	274 (94.16%)		505 (91.82%)	274 (94.16%)	
IIA1 + IIA2 + IIB	311 (20.62%)	17 (5.84%)		45 (8.18%)	17 (5.84%)	
Cell type			0.011			0.962
Squamous	1055 (69.96%)	225 (77.32%)		426 (77.45%)	225 (77.32%)	
Non-squamous	453 (30.04%)	66 (22.68%)		124 (22.55%)	66 (22.68%)	
Hysterectomy type			0.481			0.788
Type 1,2	27 (1.79%)	7 (2.41%)		15 (2.73%)	7 (2.41%)	
Type 3	1481 (98.21%)	284 (97.59%)		535 (97.27%)	284 (97.59%)	
Surgical approach			<0.001			0.509
Laparotomy	1062 (70.42%)	150 (51.55%)		294 (53.45%)	150 (51.55%)	
MIS	446 (29.58%)	141 (48.45%)		256 (46.55%)	141 (48.45%)	
Total tumor size (cm)	3.2 [0.5–11]	2.5 [0.5–12]	<0.001	3 [0.5–10.5]	2.5 [0.5–12]	0.328
Depth of invasion			<0.001			0.494
<1/2	457 (30.31%)	122 (41.92%)		218 (39.64%)	122 (41.92%)	
>1/2	1051 (69.69%)	169 (58.08%)		332 (60.36%)	169 (58.08%)	
LVSI			<0.001			0.662
Negative	706 (46.82%)	189 (64.95%)		345 (62.73%)	189 (64.95%)	
Positive	579 (38.4%)	74 (25.43%)		144 (26.18%)	74 (25.43%)	
Unknown	223 (14.79%)	28 (9.62%)		61 (11.09%)	28 (9.62%)	
Parametrial invasion			<0.001			0.509
Negative	1223 (81.1%)	262 (90.03%)		487 (88.55%)	262 (90.03%)	
Positive	285 (18.9%)	29 (9.97%)		63 (11.45%)	29 (9.97%)	
Resection margin			0.523			0.778
Negative	1473 (97.68%)	286 (98.28%)		539 (98%)	286 (98.28%)	
Positive	35 (2.32%)	5 (1.72%)		11 (2%)	5 (1.72%)	
Pelvic lymph node			<0.001			0.526
Negative	1149 (76.55%)	257 (88.32%)		475 (86.84%)	257 (88.32%)	
Positive	352 (23.45%)	34 (11.68%)		72 (13.16%)	34 (11.68%)	
Paraaortic lymph node			0.042			0.744
Negative	1468 (97.35%)	289 (99.31%)		547 (99.45%)	289 (99.31%)	
Positive	40 (2.65%)	2 (0.69%)		3 (0.55%)	2 (0.69%)	
Initial treatment			<0.001			<0.001
Surgery	644 (42.71%)	200 (68.73%)		286 (52%)	200 (68.73%)	
Surgery + RT	409 (27.12%)	40 (13.75%)		141 (25.64%)	40 (13.75%)	
Surgery + CCRT	455 (30.17%)	51 (17.53%)		123 (22.36%)	51 (17.53%)	

Data are shown in number (%) or median [range]. MIS, minimally invasive surgery; LVSI, lympho-vascular space invasion; RT, radiation therapy; CCRT, concurrent chemoradiation therapy.

**Table 2:** Multivariate analysis for progression-free survival and overall survival.

	PFS		OS	
	HR (95% CI)	*p*-Value	HR (95% CI)	*p*-Value
Conization				
Not done	1		1	
Done	0.65 (0.41–1.01)	0.056	0.59 (0.34–1.02)	0.059
Age	0.98 (0.96–1.00)	0.068	1.03 (1.01–1.05)	0.011
Cell type				
Squamous	1		1	
Non-squamous	1.92 (1.26–2.94)	0.002	2.10 (1.27–3.48)	0.004
Total tumor size (cm)	1.09 (0.97–1.21)	0.139		
Depth of invasion				
<1/2	1			
>1/2	1.54 (0.95–2.48)	0.080		
LVSI				
Negative	1			
Positive	1.98 (1.24–3.17)	0.004		
Parametrial invasion				
Negative	1		1	
Positive	1.77 (0.95–3.29)	0.072	2.10 (1.10–4.01)	0.025
Resection margin				
Negative	1			
Positive	2.71 (0.92–7.99)	0.071		
Pelvic lymph node				
Negative	1		1	
Positive	1.91 (0.98–3.72)	0.057	1.99 (1.08–3.67)	0.027
Paraaortic lymph node				
Negative	1		1	
Positive	10.86 (3.92–30.12)	<0.001	10.42 (3.43–31.64)	<0.001
Initial treatment				
Surgery	1			
Surgery + RT	0.47 (0.25–0.89)	0.020		
Surgery + CCRT	0.45 (0.22–0.95)	0.037		

PFS, progression free survival; OS, overall survival; HR, hazard ratio; CI, confidence interval; LVSI, lympho-vascular space invasion; RT, radiation therapy; CCRT, concurrent chemoradiation therapy.

## Data Availability

The datasets used and/or analyzed during the current study are available from the corresponding author on reasonable request.

## Supplementary Materials

The following supporting information can be downloaded at https://www.mdpi.com/article/10.3390/cancers14163863/s1, Table S1: Univariate and multivariate analyses for progression-free survival (all variables included), Table S2: Univariate and multivariate analyses for overall survival (all variables included), Figure S1: Kaplan–Meier curves for progression-free survival (PFS) and overall survival (OS) in subgroup of patients with tumor size < 4 cm. (A) PFS for patients with tumor size < 2 cm; (B) OS for patients with tumor size < 2 cm; (C) PFS for patients with tumor size ≥ 2 cm and < 4 cm; (D) OS for patients with tumor size ≥ 2 cm and <4 cm, Figure S2: Kaplan–Meier curves for progression-free survival (PFS) and overall survival (OS) according to resection margin of conization. (A) PFS for patients with tumor size < 2 cm; (B) OS for patients with tumor size < 2 cm; (C) PFS for patients with tumor size ≥ 2 cm and < 4 cm; (D) OS for patients with tumor size ≥ 2 cm and < 4 cm; (E) PFS for patients with tumor size ≥ 4 cm; (F) OS for patients with tumor size ≥ 4 cm.
