# Supplementary material for: The Role of Conization before Radical Hysterectomy in Cervical Cancer including High Risk Factors of Recurrence: Propensity Score Matching
Source: Cancers (Basel). 2022 Aug 10;14(16):3863. doi: 10.3390/cancers14163863 (PMC9405990; doi:10.3390/cancers14163863)

Supplementary Table S1. Univariate and multivariate analyses for progression-free survival (all variables included).

	Univariate		Mutivariate	
	**HR (95% CI)**	***p*-value**	**HR (95% CI)**	***p*-value**
Conization				
Not done	1		1	
Done	0.73 (0.48–1.13)	0.156	0.65 (0.41–1.02)	0.060
Age	0.98 (0.97–1.00)	0.083	0.98 (0.96–1.00)	0.092
Year of diagnosis				
~ 2000	1		1	
2000 ~ 2010	1.87 (0.74–4.75)	0.187	1.61 (0.60–4.29)	0.342
2010 ~	2.54 (1.01–6.38)	0.047	1.67 (0.59–4.73)	0.335
Stage				
IB1 + IB2	1		1	
IIA1 + IIA2 + IIB	1.42 (0.74–2.74)	0.289	1.03 (0.49–2.17)	0.942
Cell type				
Squamous	1		1	
Non-squamous	1.69 (1.11–2.56)	0.014	1.95 (1.27–2.99)	0.002
Hysterectomy type				
Type 1,2	1		1	
Type 3	2.77 (0.39–19.86)	0.311	2.50 (0.34–18.31)	0.368
Surgical approach				
Laparotomy	1		1	
MIS	1.27 (0.86–1.88)	0.232	1.28 (0.79–2.08)	0.316
Total tumor size (cm)	1.17 (1.07–1.28)	<0.001	1.10 (0.98–1.23)	0.121
Depth of invasion				
<1/2	1		1	
>1/2	1.66 (1.08–2.55)	0.021	1.56 (0.96–2.53)	0.073
LVSI				
Negative	1		1	
Positive	2.40 (1.60–3.59)	<0.001	2.03 (1.27–3.26)	0.003
Parametrial invasion				
Negative	1		1	
Positive	2.64 (1.66–4.20)	<0.001	1.63 (0.86–3.08)	0.131
Resection margin				
Negative	1		1	
Positive	2.15 (0.79–5.84)	0.134	2.75 (0.92–8.27)	0.071
Pelvic lymph node				
Negative	1		1	
Positive	3.16 (2.07–4.83)	<0.001	1.96 (1.00–3.85)	0.051
Paraaortic lymph node				
Negative	1		1	
Positive	21.97 (8.84–54.63)	<0.001	10.38 (3.86–28.64)	<0.001
Initial treatment				
Surgery	1		1	
Surgery + RT	0.76 (0.43–1.36)	0.363	0.49 (0.26–0.92)	0.028
Surgery + CCRT	2.09 (1.36–3.20)	0.001	0.46 (0.22–0.99)	0.047

HR, hazard ratio; CI, confidence interval; MIS, minimally invasive surgery; LVSI, lympho-vascular space invasion; RT, radiation therapy; CCRT, concurrent chemoradiation therapy.

Supplementary Table S2. Univariate and multivariate analyses for overall survival (all variables included).

	Univariate		Mutivariate	
	**HR (95% CI)**	***p*-value**	**HR (95% CI)**	***p*-value**
Conization				
Not done	1		1	
Done	0.55 (0.32–0.95)	0.031	0.57 (0.32–1.01)	0.056
Age	1.03 (1.01–1.05)	0.007	1.03 (1.00–1.05)	0.014
Year of diagnosis				
~ 2000	1		1	
2000 ~ 2010	1.28 (0.64–2.54)	0.486	1.10 (0.53–2.27)	0.806
2010 ~	1.01 (0.45–2.26)	0.985	0.59 (0.22–1.56)	0.289
Stage				
IB1 + IB2	1		1	
IIA1 + IIA2 + IIB	2.15 (1.10–4.22)	0.02	1.27 (0.59–2.73)	0.54
Cell type				
Squamous	1		1	
Non-squamous	1.74 (1.06–2.85)	0.029	2.30 (1.37–3.89)	0.002
Hysterectomy type				
Type 1,2	1		1	
Type 3	0.76 (0.19–3.12)	0.706	0.89 (0.20–3.88)	0.874
Surgical approach				
Laparotomy	1		1	
MIS	0.72 (0.42–1.25)	0.245	0.98 (0.50–1.91)	0.954
Total tumor size (cm)	1.14 (1.02–2.98)	0.018	0.97 (0.84–1.12)	0.691
Depth of invasion				
<1/2	1		1	
>1/2	1.75 (1.02–2.98)	0.041	1.31 (0.71–2.43)	0.387
LVSI				
Negative	1		1	
Positive	1.99 (1.21–3.28)	0.007	1.51 (0.84–2.73)	0.172
Parametrial invasion				
Negative	1		1	
Positive	3.34 (1.88–5.92)	<0.001	1.81 (0.87–3.75)	0.111
Resection margin				
Negative	1		1	
Positive	2.02 (0.63–6.46)	0.234	1.87 (0.52–6.68)	0.335
Pelvic lymph node				
Negative	1		1	
Positive	2.96 (1.77–4.94)	<0.001	1.45 (0.67–3.14)	0.344
Paraaortic lymph node				
Negative	1		1	
Positive	17.73 (6.40–49.14)	<0.001	11.68 (3.67–37.15)	<0.001
Initial treatment				
Surgery	1		1	
Surgery + RT	1.40 (0.76–2.56)	0.278	0.83 (0.41–1.68)	0.614
Surgery + CCRT	3.11 (1.82–5.32)	<0.001	1.26 (0.54–2.95)	0.597

HR, hazard ratio; CI, confidence interval; MIS, minimally invasive surgery; LVSI, lympho-vascular space invasion; RT, radiation therapy; CCRT, concurrent chemoradiation therapy.

Supplementary Figure S1. Kaplan-Meier curves for progression-free survival (PFS) and overall survival (OS) in subgroup of patients with tumor size < 4 cm. (**A**) PFS for patients with tumor size < 2 cm; (**B**) OS for patients with tumor size < 2 cm; (**C**) PFS for patients with tumor size ≥ 2 cm and < 4 cm; (**D**) OS for patients with tumor size ≥ 2 cm and < 4 cm.

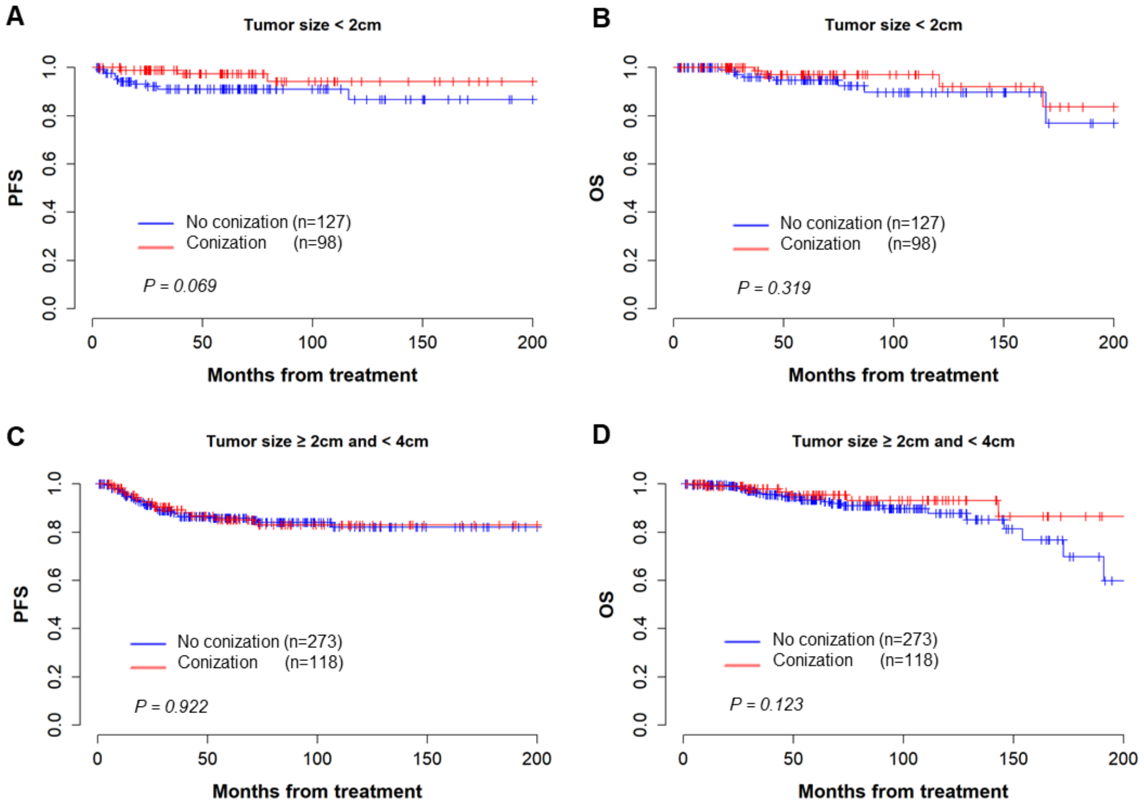

Supplementary Figure S2. Kaplan-Meier curves for progression-free survival (PFS) and overall survival (OS) according to resection margin of conization. (**A**) PFS for patients with tumor size < 2 cm; (**B**) OS for patients with tumor size < 2 cm; (**C**) PFS for patients with tumor size ≥ 2 cm and < 4 cm; (**D**) OS for patients with tumor size ≥ 2 cm and < 4 cm; (**E**) PFS for patients with tumor size ≥ 4 cm; (**F**) OS for patients with tumor size ≥ 4 cm.

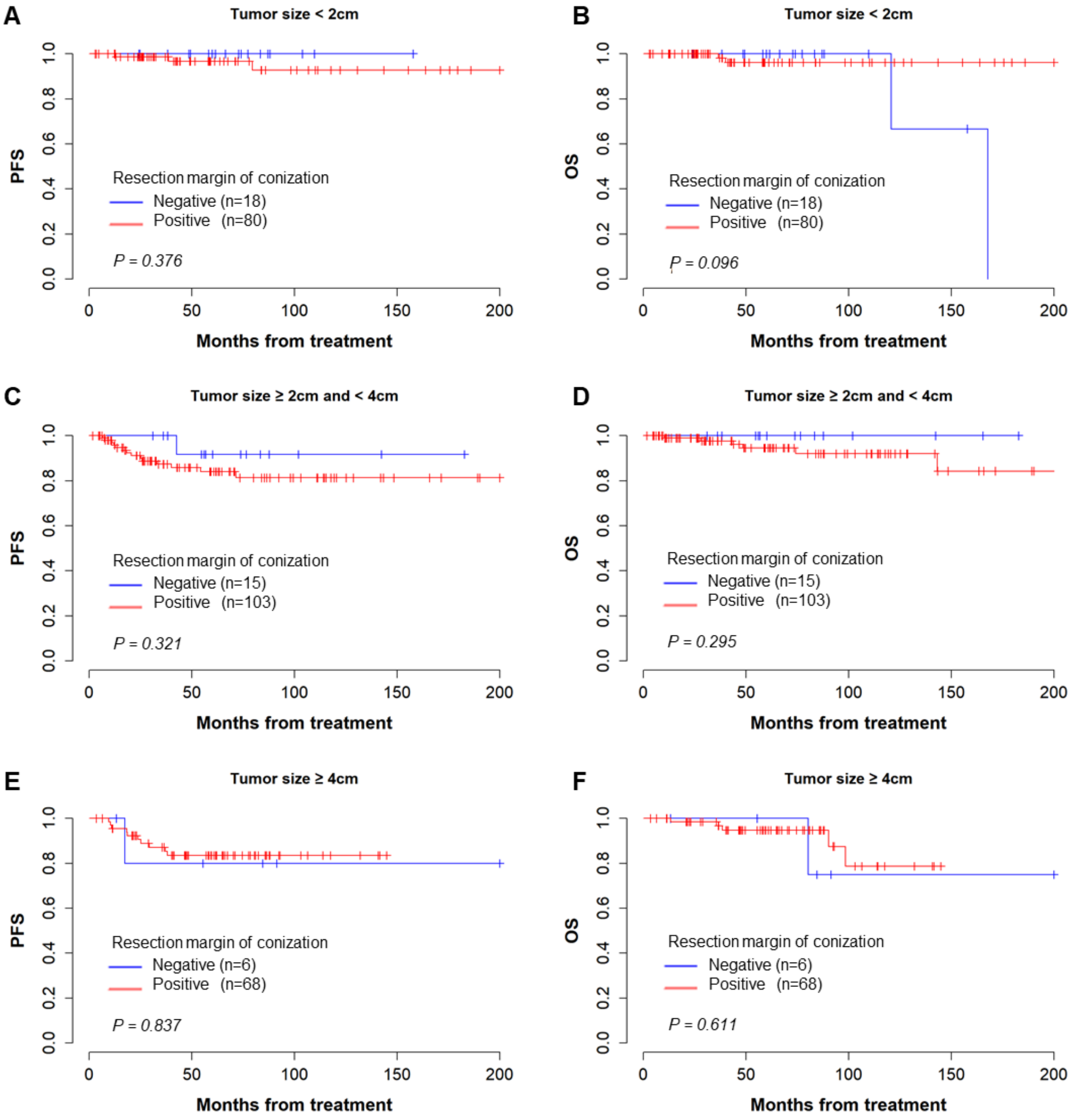

Supplement: Supplementary file 1 [file cancers-14-03863-s001.zip › cancers-1807148-supplementary.pdf]
